# Avaliação da influência de alterações cardíacas na ultrassonografia vascular periférica de idosos

**DOI:** 10.1590/1677-5449.010015

**Published:** 2016

**Authors:** Alcides José Araújo Ribeiro, Andréa Campos de Oliveira Ribeiro, Márcia Marisia Maciel Rodrigues, Sandra de Barros Cobra Negreiros, Ana Cláudia Cavalcante Nogueira, Osório Luís Rangel Almeida, José Carlos Quináglia e Silva, Ana Patrícia de Paula

**Affiliations:** 1 Hospital de Base do Distrito Federal – HBDF, Unidade de Cirurgia Vascular e Angiologia, Brasília, DF, Brasil.; 2 Clínica Villas Boas, Brasília, DF, Brasil.; 3 Hospital de Base do Distrito Federal – HBDF, Unidade de Cardiologia, Brasília, DF, Brasil.; 4 Hospital de Base do Distrito Federal – HBDF, Brasília, DF, Brasil.

**Keywords:** ultrassonografia Doppler, cardiopatias, diagnóstico, insuficiência da valva aórtica, veia femoral, fluxo pulsátil

## Abstract

**Contexto:**

As cardiopatias podem causar alterações no formato das ondas da ultrassonografia vascular (UV) em vasos periféricos. Essas alterações, tipicamente bilaterais e sistêmicas, são pouco conhecidas e estudadas.

**Objetivo:**

Avaliar as ondas periféricas da UV de pacientes idosos para identificar alterações decorrentes de cardiopatias.

**Métodos:**

Foram estudados 183 pacientes idosos submetidos a UV periférica no ano de 2014.

**Resultados:**

Foram avaliados 102 mulheres (55,7%) e 81 homens (44,3%) com idade entre 60 e 91 anos (média de 70,4±7,2 anos). Encontraram-se alterações pela UV em 84 pacientes (45,9%). Foram identificadas 138 alterações de oito dos 13 tipos descritos na literatura: arritmia, onda *bisferiens* de pico sistólico, baixa velocidade de pico sistólico, pulsatilidade em veias femorais, bradicardia, taquicardia, onda de pulso *parvus tardus* e onda de pulso *alternans*. Houve baixa concordância entre a presença e a não presença de alterações na UV e na avaliação cardiológica. Na análise específica das alterações, os exames tiveram uma concordância variável, que foi boa para o achado de taquicardia, moderada para arritmia e baixa para bradicardia. Não houve concordância entre a UV e os exames cardiológicos para as demais alterações.

**Conclusões:**

É possível identificar determinadas alterações cardíacas em idosos por meio da análise do formato das ondas periféricas da UV. É importante reconhecer e relatar a presença dessas alterações, pela possibilidade de alertar para um diagnóstico ainda não identificado nesses pacientes. Entretanto, mais estudos são necessários para que seja definida a importância das alterações no formato das ondas Doppler periféricas no reconhecimento de cardiopatias.

## INTRODUÇÃO

Segundo o governo federal brasileiro, as doenças cardiovasculares são responsáveis por 29,4% do total de mortes registradas a cada ano, o que coloca o Brasil entre os países com maiores índices de mortes por essas doenças^1^.

As cardiopatias são frequentes em pacientes idosos submetidos a UV. Portanto, a interpretação das ondas Doppler periféricas nesses pacientes deve considerar que a função cardíaca pode ser anormal, o que pode levar a alterações nessas ondas espectrais nos exames periféricos^2^.

A avaliação do fluxo por meio da UV deve levar em consideração aspectos da fisiologia cardiovascular, entre eles o ritmo e a função cardíaca e os parâmetros associados a pré e pós-cargas. Alterações no ritmo cardíaco e na função sistólica e/ou diastólica, presença de valvulopatias e as condições hemodinâmicas em que a UV foi realizada devem ser consideradas na interpretação dos padrões de fluxo^3^^,^ 4.

Em 1985, foram publicados os padrões normais das ondas periféricas da UV. Posteriormente, foram reconhecidos outros padrões para as doenças vasculares^5^.

As possíveis alterações por efeito cardíaco no formato das ondas da UV em exames periféricos, que, por definição, tendem a ser sistêmicas e bilaterais, não são amplamente conhecidas e divulgadas. Na grande maioria das vezes, não são reconhecidas durante a realização do exame e, quando o são, o examinador as ignora e não as relata no laudo.

**Figura 1:**
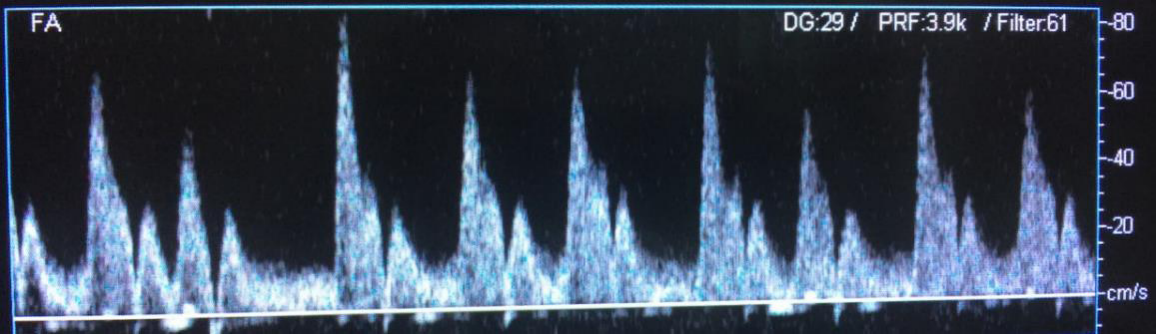
Fibrilação atrial.

**Figura 2:**
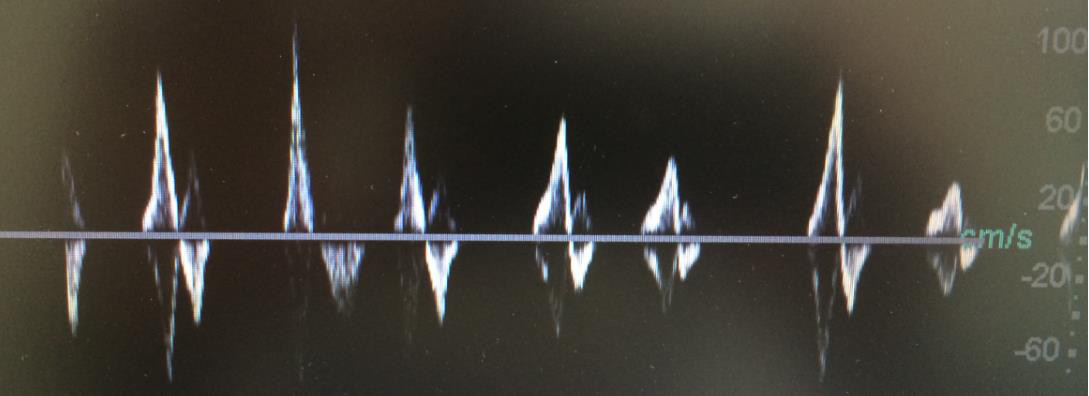
Fibrilação atrial.

**Figura 3:**
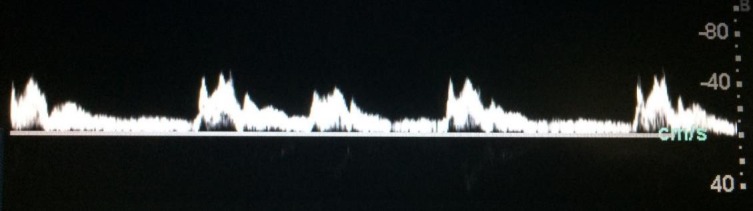
Onda *bisferiens* e extrassístole.

**Figura 4:**
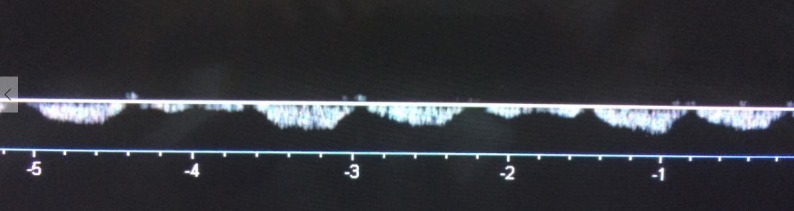
Pulsatilidade em veia femoral.

**Figura 5:**
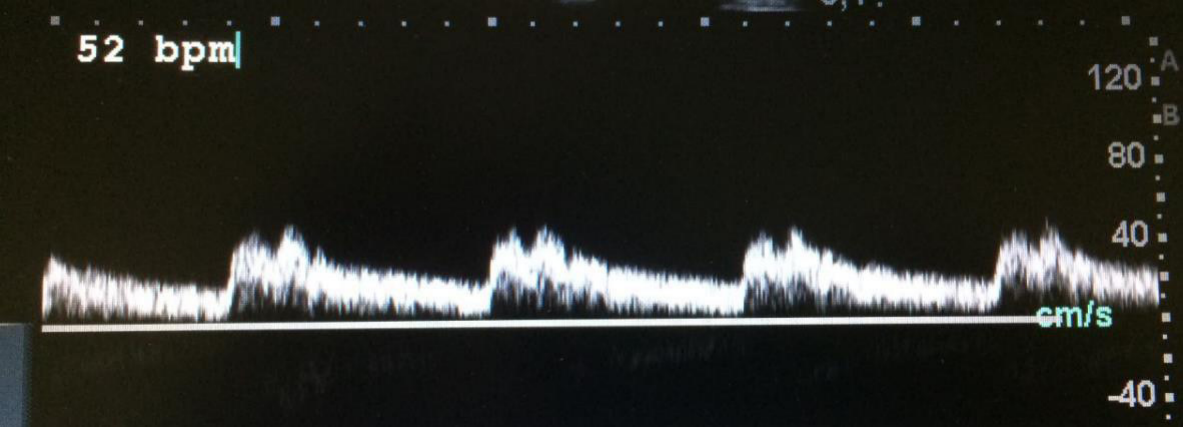
Onda *bisferiens* e bradicardia.

As alterações de origem cardíaca nos formatos das ondas periféricas da UV descritas na literatura são: arritmia^2^^,^ 6 ^,^ 7 (Figuras 1, 2 e 3), pulsatilidade na veia femoral comum e veia poplítea^2^^,^ 8 (Figura 4), onda *bisferiens* de pico sistólico^6^^,^ 9 ^-^ 11 (Figuras 3 e 5), baixa velocidade de pico sistólico^1^, bradicardia^2^ (Figura 5), taquicardia^2^, onda de pulso *alternans* 12, onda de pulso *parvus tardus* 11 ^-^ 13, onda de pulso em martelo d’água^9^, pico e cúpula sistólica^13^, aumento na velocidade de pico sistólico devido ao débito cardíaco alto^10^, onda paradoxa^14^ e ondas causadas por dispositivos de assistência cardíaca^11^^,^ 12 ^,^ 15.

O objetivo deste estudo foi avaliar a presença dessas alterações espectrais nas ondas da UV de pacientes idosos submetidos a ecografia vascular arterial e/ou venosa e verificar a concordância dos achados com os diagnósticos e exames cardiológicos.

## MÉTODOS

O estudo foi realizado no Hospital de Base do Distrito Federal (HBDF), Brasília, DF, Brasil, no período de dezembro de 2013 a dezembro de 2014, após aprovação pelo Comitê de Ética. Trata-se de um estudo transversal e analítico, com amostra composta por 183 pacientes idosos.

Critério de inclusão: pacientes com idade acima de 60 anos submetidos a UV periférica que tinham condições de fornecer as informações necessárias e de comparecer às avaliações cardiológicas quando solicitadas e que assinaram o termo de consentimento livre e esclarecido. Critérios de exclusão: pacientes com instabilidade hemodinâmica e/ou que não apresentavam condições de fornecer as informações necessárias e de comparecer às avaliações cardiológicas quando solicitadas. Os pacientes foram divididos em dois grupos. O grupo I era composto de 133 pacientes sem cardiopatia previamente diagnosticada, entre os quais 72 (54,13%) já estavam agendados para a realização de UV e foram convidados a comparecer para a realização do exame, e 61 (45,87%) não possuíam solicitação para realização do exame e foram convidados a participar do estudo durante avaliação em ambulatórios de medicina interna do HBDF, quando então foram encaminhados para realizar exame de *screening* de carótidas. O grupo II incluiu 50 pacientes com diagnóstico prévio de cardiopatia realizado por cardiologista. Os exames ecográficos nos dois grupos foram realizados sem que o examinador conhecesse a história patológica pregressa dos pacientes. Os pacientes do grupo I foram encaminhados para avaliação cardiológica após a realização da UV, exceto se comprovassem consulta cardiológica nos últimos 30 dias. O cardiologista avaliador desconhecia o resultado da UV e, quando necessário, solicitava exames complementares da rotina cardiológica na unidade de cardiologia do HBDF. Todos os pacientes do estudo fizeram eletrocardiograma.

### Protocolo ecográfico

Os dispositivos para UV usados no estudo foram um aparelho da marca Toshiba modelo Aplio 50® (Toshiba, Japão), um aparelho Sonosite M-Turbo® (Sonosite, EUA) e um aparelho Aloka SSD-1700 DYNAVIEW II® (Aloka, Japão) com transdutor linear de 4 a 7 MHz e gel não aquecido. Todos os exames foram realizados por um especialista em angiologia e cirurgia vascular com certificado de atuação em ecografia vascular com Doppler. O exame foi feito com o paciente em decúbito dorsal, a perna em posição passiva e rotação neutra, leve flexão do joelho e rotação contralateral da cabeça ao lado a ser estudado. Foram considerados os formatos espectrais das ondas Doppler em seis artérias (artérias carótidas comuns direita e esquerda, artérias braquiais direita e esquerda e artérias femorais comuns direita e esquerda) para avaliação da bilateralidade e a presença de localização arterial sistêmica, e as veias femorais comuns direita e esquerda para avaliação da repercussão cardíaca.

### Estatística

A análise descritiva da amostra foi feita por meio da média e do desvio-padrão da idade e da frequência dos demais dados. Para avaliar a concordância entre a UV e os exames cardiológicos, utilizou-se o índice de concordância Kappa. Foi usada a classificação proposta por Landis & Kopp, na qual a variação da concordância entre 0 e 0,2 é considerada insignificante; entre 0,21 e 0,40 é baixa; entre 0,41 e 0,60 é moderada; entre 0,61 e 0,80 é boa; e entre 0,81 e 1,00 é excelente^16^^,^ 17.

As análises estatísticas deste trabalho foram realizadas com o auxílio do programa IBM SPSS Statistics 20® (Statistical Package for the Social Sciences, Chicago, EUA).

## RESULTADOS

Foram avaliados 183 pacientes (102 mulheres e 81 homens), com idade entre 60 e 91 anos e média de 70,4 (±7,2) anos. A maioria dos pacientes (57,4%) não apresentava história de cardiopatia prévia. Foram encontradas oito das 13 alterações descritas na literatura^2^^-^ 15. Encontraram-se alterações na UV em 84 pacientes (45,9%), dos quais 54 (40,6%) eram do grupo I e 30 (60%) do grupo II. No grupo I, houve maior número de alterações nos pacientes que já vieram com pedidos de exames para diversas patologias (63,6%) em comparação com os pacientes que foram convidados para o estudo (36,36%). As 138 alterações encontradas estão descritas na Tabela 1. Uma única alteração no formato da onda Doppler foi observada em 62 pacientes (72,94%), duas alterações em 13 (15,29%), três alterações em sete (8,23%) e quatro alterações em dois (2,35%). Apenas 24 pacientes submetidos ao exame de UV não concluíram a avaliação cardiológica e, portanto, não foram considerados para os testes de concordância. Houve baixa concordância entre a presença e a não presença de alterações na UV e na avaliação cardiológica (Kappa = 0,251). Para o grupo II, a concordância foi insignificante (Kappa = 0,109), pois quase todos os pacientes apresentaram alguma alteração na avaliação cardiológica, enquanto na UV, apenas metade dos pacientes apresentou alteração. Sessenta por cento dos pacientes que apresentaram alterações nos formatos de onda Doppler tinham história de cardiopatia prévia. Na análise isolada das alterações, os exames tiveram uma concordância variável, que foi boa para o achado de taquicardia (Kappa = 0,66), moderada para arritmia (Kappa = 0,494) e baixa para bradicardia (Kappa = 0,264). Com relação às demais alterações, não houve concordância entre a UV e os exames cardiológicos (Tabela 2), mas quando se considerou a concordância entre as alterações da UV e os outros diagnósticos e achados cardiológicos relacionados à alteração estudada, houve um aumento de concordância para onda de pulso *parvus tardus*, onda *bisferiens* e onda *alternans* (Tabela 3). A concordância da onda *bisferiens* e da onda *parvus tardus* com os achados cardiológicos foi considerada insignificante (Kappa = 0,135 e 0,104, respectivamente), mas quando a onda *bisferiens* foi correlacionada com a insuficiência aórtica, e a esclerose valvar aórtica foi correlacionada com a onda *parvus tardus*, a concordância aumentou, passando de insignificante para baixa (Kappa = 0,224 e 0,265, respectivamente). Quando avaliada a onda de pulso *alternans*, não houve concordância entre as alterações encontradas na UV e na avaliação cardiológica, mas quando foi correlacionada com outros diagnósticos e achados cardiológicos, a concordância passou para insignificante (Kappa = 0,003). Não se evidenciaram: onda de pulso em martelo d’água, pico e cúpula sistólica, aumento na velocidade de pico sistólico devido ao débito cardíaco alto, onda paradoxa e ondas causadas por dispositivos de assistência cardíaca.

**Tabela 1:** Alterações no formato de ondas Doppler.

**Alterações**	**N (%)**
Arritmia	38 (27,5)
Onda de pulso *bisferiens*	24 (17,4)
Baixa velocidade de pico sistólico	22 (15,9)
Pulsatilidade em veias femorais	21 (15,2)
Bradicardia	11 (8,0)
Taquicardia	9 (6,5)
Onda de pulso *parvus tardus*	7 (5,1)
Onda de pulso *alternans*	6 (4,3)

**Tabela 2:** Concordância entre os exames eco-Doppler e diagnósticos cardiológicos.

**Sintoma**	**eco-Doppler** **(n = 159)**	**Diagnóstico cardiológico** **(n = 159)**	**Kappa**
Arritmia	21,4%	17,0%	0,494
Pulsatilidade em veia femoral	11,3%	5,0%	0,098
Onda *bisferiens*	12,6%	9,4%	0,135
Baixa velocidade de pico sistólico	5,7%	4,4%	0,079
Bradicardia	6,9%	1,9%	0,264
Taquicardia	1,9%	1,9%	0,66
Pulso *alternans*	2,5%	0,0%	-
Fluxo *parvus tardus*	5,0%	3,8%	0,104
Fluxo em martelo d'água	0,0%	0,0%	-

**Tabela 3:** Concordância entre os exames e os grupos de outros achados cardiológicos.

**Sintoma**	**eco-Doppler** **(n = 159)**	**Exames cardiológicos** **(n = 159)**	**Kappa**
Arritmia/cardiopatia chagásica/trombo cardíaco	21,4%	20,1%	0,464
Pulsatilidade em veia femoral/insuficiência tricúspide	11,3%	25,8%	0,175
Onda *bisferiens*/insuficiência aórtica	12,6%	23,3%	0,224
Baixa velocidade de pico sistólico/isquemia miocárdica/baixa fração de ejeção/hipocontratilidade/estenoses coronarianas/hipoperfusão miocárdica	5,7%	24,5%	-0,010
Bradicardia	6,9%	1,9%	0,264
Taquicardia	1,9%	1,9%	0,66
Pulso a*lternans*/isquemia miocárdica/baixa fração de ejeção/estenoses coronarianas/hipoperfusão	2,5%	23,3%	0,003
Fluxo *parvus tardus*/esclerose valvar aórtica	3,8%	12,6%	0,265
Fluxo em martelo d'água	0,0%	23,3%	-

## DISCUSSÃO

O presente estudo demonstra que a análise do formato das ondas Doppler periféricas em idosos pode sugerir o diagnóstico de alterações ou doenças cardíacas. Segundo o nosso conhecimento, não há estudo semelhante na literatura comparando os achados nas ondas Doppler com a avaliação cardiológica, e este estudo é o primeiro a avaliar todas as 13 alterações já descritas. A concordância geral entre a presença e a não presença de alterações na UV e na avaliação cardiológica foi baixa. O número de alterações encontradas foi maior no grupo dos pacientes cardiopatas em relação aos pacientes do grupo sem cardiopatia previamente diagnosticada (60% *versus* 41,35%), como era esperado. Tanto na UV quanto na avaliação cardiológica, houve um maior número de alterações nos pacientes com história prévia de cardiopatias. A concordância específica das alterações encontradas na UV com a avaliação cardiológica foi variável, sendo maior para taquicardia, arritmia e bradicardia. Nas outras alterações, não houve concordância inicial entre a UV e os exames realizados, mas quando se considerou outros diagnósticos e achados cardiológicos encontrados e que possuíam relação com as alterações específicas estudadas, houve um aumento de concordância para a onda de pulso *parvus tardus*, onda *bisferiens* e onda *alternans.* Resultados não significantes entre os grupos podem ser decorrência de uma amostra pequena. Considerando-se a possibilidade de identificar alterações no formato das ondas Doppler durante o exame de UV, o paciente idoso poderá dispor de mais um mecanismo investigador de saúde cardiovascular e ainda ter a oportunidade de identificar relevantes alterações cardíacas, grande causa de morte nessa faixa etária.

## CONCLUSÃO

O formato das ondas Doppler periféricas permite detectar achados semiológicos ou diagnósticos da propedêutica cardíaca. Existem correlações variáveis entre as alterações no formato das ondas Doppler periféricas e o diagnóstico cardiológico. A melhor concordância foi com taquicardia, seguida por arritmia e bradicardia. As demais correlações não demonstraram significância.

Essas alterações deveriam ser relatadas no laudo da UV, o que auxiliaria o diagnóstico pelo médico assistente. Entretanto, mais estudos são necessários para que seja definida a importância das alterações no formato das ondas Doppler periféricas no reconhecimento de cardiopatias.
