# Supplementary material for: Avaliação da influência de alterações cardíacas na ultrassonografia vascular periférica de idosos
Source: J Vasc Bras. 2016 Jul-Sep;15(3):205–9. [Article in Portuguese] doi: 10.1590/1677-5449.010015 (PMC5829757; doi:10.1590/1677-5449.010015)
Supplement: Translation

# Translation: Assessment of cardiac influence on peripheral vascular Doppler in the elderly

**Authors:** Alcides José Araújo Ribeiro, Andréa Campos de Oliveira Ribeiro, Márcia Marisia Maciel Rodrigues, Sandra de Barros Cobra Negreiros, Ana Cláudia Cavalcante Nogueira, Osório Luís Rangel Almeida, José Carlos Quináglia e Silva, Ana Patrícia de Paula

**Affiliations:** 1 Hospital de Base do Distrito Federal – HBDF, Unidade de Cirurgia Vascular e Angiologia, Brasília, DF, Brazil.; 2 Clínica Villas Boas, Brasília, DF, Brazil.; 3 Hospital de Base do Distrito Federal – HBDF, Unidade de Cardiologia, Brasília, DF, Brazil.; 4 Hospital de Base do Distrito Federal – HBDF, Brasília, DF, Brazil.

**Keywords:** Doppler ultrasonography, heart diseases, diagnosis, aortic valve insufficiency, vein femoral, pulsating flow

## Abstract

**Background:**

Heart diseases can cause changes to vascular ultrasonography (VUS) waveforms in peripheral vessels. These changes are typically bilateral and systemic, they have been little studied, and little is known about them.

**Objective:**

To assess peripheral VUS waveforms in elderly patients in order to identify changes caused by heart diseases.

**Methods:**

During 2014, a total of 183 elderly patients were examined with peripheral VUS and the results were analyzed.

**Results:**

The sample comprised 102 women (55.7%) and 81 men (44.3%) with ages ranging from 60 to 91 years (mean of 70.4±7.2 years). Abnormalities were identified in VUS waveforms in 84 patients (45.9%). A total of 138 abnormalities were identified and classified into eight of the 13 categories described in the literature, as follows: arrhythmia, systolic pulsus bisferiens, low peak systolic velocity, pulsatile flow in femoral veins, bradycardia, tachycardia, pulsus tardus et parvus and pulsus alternans. There was low agreement between presence/absence of VUS abnormalities and cardiological assessments. Analysis of specific abnormalities revealed variable levels of agreement between VUS and cardiological assessments, ranging from good for tachycardia, moderate for arrhythmia, to low for bradycardia. There was no agreement between VUS and cardiological examinations for the remaining categories of abnormalities.

**Conclusions:**

Certain cardiac abnormalities can be identified in elderly patients by analysis of peripheral VUS waveforms. It is important to recognize and report the presence of these abnormalities because there is a possibility that they may serve to signal hitherto unidentified diagnoses in these patients. However, further studies are needed to determine the importance of changes to peripheral Doppler waveforms to recognition of heart diseases.

## INTRODUCTION

According to the federal government of Brazil, cardiovascular diseases are responsible for 29.4% of all deaths recorded each year, which places Brazil among the countries with the highest mortality rates due to these diseases.^1^

Heart diseases are common among elderly patients who undergo VUS. As such, when interpreting peripheral Doppler waveforms from these patients, the fact that cardiac function could be abnormal should be taken into account, since this could cause abnormalities in the spectral waves of peripheral examinations.^2^

Vascular ultrasound flow assessments should take into account elements of cardiovascular physiology, including cardiac rhythm and function and parameters associated with preload and afterload. Changes to cardiac rhythm and systolic and/or diastolic function, presence of valve disease, and the hemodynamic conditions under which VUS was conducted should also be considered when interpreting flow patterns.^3^^,^ 4

Standards for normal peripheral VUS waves were published in 1985. Later, other patterns related to vascular diseases were recognized.^5^

By definition, the possible changes to peripheral VUS examination waveforms resulting from cardiac effects tend to be systemic and bilateral, but they are neither well publicized nor well known. In the great majority of cases these signs go unnoticed during examinations and when examiners do recognize them they very often ignore them and do not include them in their examination reports.

The following cardiac-induced changes to peripheral VUS waveforms are described in the literature: arrhythmia^2^^,^ 6 ^,^ 7 (Figures 1, 2 and 3), pulsatile waves in the common femoral and popliteal veins^2^^,^ 8 (Figure 4), systolic pulsus bisferiens^6^^,^ 9 ^-^ 11 (Figures 3 and 5), low peak systolic velocity,^1^ bradycardia^2^ (Figure 5), tachycardia,^2^ pulsus alternans,^12^ pulsus tardus et parvus,^11^^-^ 13 water hammer pulse,^9^ systolic spike and dome,^13^ elevated peak systolic velocity caused by high cardiac output,^10^ pulsus paradoxus,^14^ and waves caused by cardiac assistance devices.^11^^,^ 12 ^,^ 15

**Figure 1:** Atrial fibrillation.

**Figure 2:** Atrial fibrillation.

**Figure 3:** Pulsus bisferiens and extrasystole.

**Figure 4:** Pulsatile waves in the femoral vein.

**Figure 5:** Pulsus bisferiens and bradycardia.

The objectives of this study were to evaluate the presence of these spectral changes in VUS waveforms from elderly patients who underwent vascular arterial and/or venous echography and to analyze the degree of agreement between these findings and cardiological examinations and diagnoses.

## METHODS

The study was conducted at the Hospital de Base do Distrito Federal (HBDF), in Brasília, DF, Brazil, from December 2013 to December 2014, after prior approval from the Ethics Committee. It is a cross-sectional and analytical study of a sample comprising 183 elderly patients.

Inclusion criteria: patients aged over 60 years, who underwent peripheral VUS, who were capable of providing the information needed, who were able to attend for cardiological assessments when requested, and who signed a free and informed consent form. Exclusion criteria: patients with hemodynamic instability, and/or who were unable to provide the information needed and attend for cardiological assessments when requested. These patients were subdivided into two subsets. Group I comprised 133 patients without prior diagnoses of heart disease, 72 (54.13%) of whom had already been scheduled for VUS and were invited to attend for examinations and 61 (45.87%) of whom who had not been scheduled for VUS, but were invited to take part in the study during assessments at HBDF internal medicine clinics, when they were referred for carotid screening tests. Group II comprised 50 patients with prior diagnoses of heart disease made by a cardiologist. For both groups, the examiner who performed the ultrasound examinations was unaware of the patients’ prior medical histories. Patients in group I were referred for cardiological assessment after their VUS examinations unless they provided evidence of a cardiological consultation during the preceding 30 days. The cardiology consultant was unaware of the VUS results and when necessary requested routine supplementary cardiological tests, conducted at the HBDF cardiology unit. Electrocardiograms were conducted for all patients in the study.

### Ultrasonography protocol

The following devices were used for VUS: a Toshiba Aplio 50® (Toshiba, Japan), a Sonosite M-Turbo® (Sonosite, United States), and an Aloka SSD-1700 DYNAVIEW II® (Aloka, Japan), with a linear 4 to 7 MHz transducer and unheated gel. All examinations were conducted by a specialist in angiology and vascular surgery, certified to practice Doppler vascular echography. Examinations were conducted with patients in decubitus dorsal, the leg in a passive position at neutral rotation, slight flexion of the knee and rotation of the head contralateral to the side being studied. The spectral patterns of the Doppler waves were analyzed for six arteries (right and left common carotid arteries, right and left brachial arteries, and right and left common femoral arteries) in order to enable assessment of bilateralism and presence of systemic arterial involvement, and the right and left common femoral veins to enable assessment of cardiac repercussions.

### Statistical analysis

The sample was described statistically in terms of mean and standard deviation for age and in terms of frequencies for all other data. Agreement between the results of VUS and of cardiological tests was assessed using the Kappa index of agreement. This was categorized according to the Landis & Kopp classification, in which agreement ranging from 0 to 0.2 is considered insignificant; from 0.21 to 0.40 is low; from 0.41 to 0.60 is moderate; from 0.61 to 0.80 is good; and agreement indices in the range 0.81 to 1.00 are excellent.^16^^,^ 17

The statistical analyses in this study were conducted using IBM SPSS 20® (Statistical Package for the Social Sciences, Chicago, United States).

## RESULTS

A total of 183 patients (102 women and 81 men) aged from 60 to 91 years, with a mean age of 70.4 (±7.2) years, were assessed. The majority of these patients (57.4%) did not have a prior history of heart disease. Eight of the 13 abnormal VUS findings described in the literature were detected.^2^^-^ 15 Abnormal VUS waveforms were found in 84 patients (45.9%), 54 (40.6%) of whom were in group I and 30 (60%) of whom were in group II. Within group I, there was a larger number of abnormal findings among patients for whom tests for a range of different pathologies had already been requested (63.6%) than among those patients who had been invited to take part at the clinic (36.36%). The 138 abnormal findings are listed in Table 1. Just one abnormality of the Doppler waveforms was observed in 62 of the patients (72.94%), while 13 (15.29%) patients had two abnormalities, seven (8.23%) patients had three, and two (2.35%) patients had four abnormal findings. Just 24 of the patients who underwent the VUS examination did not complete their cardiological assessments and were therefore excluded from the analysis of agreement. There was a low level of agreement between presence/absence of abnormal VUS findings and the results of cardiological assessment (Kappa = 0.251). Agreement for group II was insignificant (Kappa = 0.109), because almost all of the patients exhibited some type of abnormality on cardiological assessment, whereas VUS only detected abnormalities in half of the patients. Sixty percent of the patients who exhibited Doppler waveform abnormalities had a prior history of heart disease. When agreement for each type of abnormality was analyzed separately the level of agreement varied, with good agreement for tachycardia (Kappa = 0.66), moderate for arrhythmia (Kappa = 0.494), and low for bradycardia (Kappa = 0.264). There was no agreement between the other VUS waveform abnormalities and cardiological test results (Table 2), but when agreement between VUS abnormalities and other cardiological findings and diagnoses related to the abnormality in question, there was a greater level of agreement for pulsus tardus et parvus, pulsus bisferiens and pulsus alternans waves (Table 3). The levels of agreement between cardiac findings and the pulsus bisferiens and pulsus tardus et parvus waves were considered insignificant (Kappa = 0.135 and 0.104, respectively), but the correlations between pulsus bisferiens and aortic insufficiency, and between pulsus tardus et parvus and aortic valve sclerosis were higher, increasing from insignificant to low (Kappa = 0.224 and 0.265, respectively). There was no agreement between VUS findings of pulsus alternans and cardiological assessment, but when correlated with other cardiological findings and diagnoses, agreement increased to insignificant (Kappa = 0.003). The following abnormalities were not detected: water hammer pulse, systolic spike and dome, elevated peak systolic velocity due to high cardiac output, pulsus paradoxus, and waves caused by cardiac assistance devices.

**Table 1:** Abnormalities of Doppler waveforms.

**Abnormalities**	**N (%)**
Arrhythmia	38 (27.5)
Pulsus bisferiens	24 (17.4)
Low peak systolic velocity	22 (15.9)
Pulsating flow in femoral veins	21 (15.2)
Bradycardia	11 (8.0)
Tachycardia	9 (6.5)
Pulsus tardus et parvus	7 (5.1)
Pulsus alternans.	6 (4.3)

**Table 2:** Agreement between Doppler ultrasonography findings and cardiological diagnoses.

**Symptom**	**Doppler ultrasonography** **(n = 159)**	**Cardiological diagnosis ** **(n = 159)**	**Kappa**
Arrhythmia	21.4%	17.0%	0.494
Pulsating flow in femoral vein	11.3%	5.0%	0.098
Pulsus bisferiens	12.6%	9.4%	0.135
Low peak systolic velocity	5.7%	4.4%	0.079
Bradycardia	6.9%	1.9%	0.264
Tachycardia	1.9%	1.9%	0.66
Pulsus alternans.	2.5%	0.0%	-
Pulsus tardus et parvus	5.0%	3.8%	0.104
Water hammer flow	0.0%	0.0%	-

**Table 3:** Agreement between tests and groups of additional cardiological findings.

**Symptom**	**Doppler ultrasonography** **(n = 159)**	**Cardiological tests** **(n = 159)**	**Kappa**
Arrhythmia/Chagas heart disease/cardiac thrombus	21.4%	20.1%	0.464
Pulsating flow in femoral vein/tricuspid insufficiency	11.3%	25.8%	0.175
Pulsus bisferiens/aortic insufficiency	12.6%	23.3%	0.224
Low peak systolic velocity/myocardial ischemia/low ejection fraction/hypocontractility/coronary stenosis/ myocardial hypoperfusion	5.7%	24.5%	-0.010
Bradycardia	6.9%	1.9%	0.264
Tachycardia	1.9%	1.9%	0.66
Pulsus alternans/myocardial ischemia/low ejection fraction/coronary stenosis/hypoperfusion	2.5%	23.3%	0.003
Pulsus tardus et parvus/aortic valve sclerosis	3.8%	12.6%	0.265
Water hammer flow	0.0%	23.3%	-

## DISCUSSION

This study demonstrates that analysis of peripheral Doppler waveforms from elderly patients can suggest diagnoses of cardiac diseases or abnormalities. As far as we are aware, there are no similar studies in the literature, comparing Doppler waveform findings with cardiological assessment and this study is the first to investigate all 13 of the abnormalities that have been described to date. The overall agreement between presence/absence of VUS abnormalities and cardiological assessment results was low. The number of abnormalities found was higher in the subset of cardiac disease patients than in the subset of patients with no previous diagnosis of heart disease (60% vs. 41.35%), as was expected. There were higher numbers of both abnormal VUS findings and of abnormal cardiological assessment findings among the patients with prior history of heart diseases. Agreement between each specific type of VUS abnormality and cardiological assessment results was variable, with higher levels of agreement for tachycardia, arrhythmia, and bradycardia. There was initially no agreement between VUS and the cardiological tests for the other types of VUS abnormality, but when other cardiological findings and diagnoses that had a relationship with the specific abnormalities in question were taken into account, the levels of agreement for pulsus tardus et parvus, pulsus bisferiens and pulsus alternans waves increased. The non-significant results between groups may be because of the small sample size. The possibility of identifying abnormalities in Doppler waveforms during VUS examinations may offer one more mechanism for investigating the cardiovascular health of elderly patients and also offer the opportunity of identifying relevant cardiac abnormalities, which are a major cause of death in this age group.

## CONCLUSIONS

Peripheral Doppler waveforms can enable detection of findings indicative of diagnoses or useful for cardiac workup. The correlations between abnormal peripheral Doppler waveforms and cardiological diagnosis vary. The best level of agreement was observed for tachycardia, followed by arrhythmia and bradycardia. Other correlations were not significant.

These abnormalities should be detailed on VUS reports, which will help the treating physician to arrive at a diagnosis. However, more studies are needed to determine the importance of abnormalities of peripheral Doppler waveforms to recognition of heart diseases.
